# Design and Evaluation of Europium Containing Mesoporous Bioactive Glass Nanospheres: Doxorubicin Release Kinetics and Inhibitory Effect on Osteosarcoma MG 63 Cells

**DOI:** 10.3390/nano8110961

**Published:** 2018-11-21

**Authors:** Ying Zhang, Meng Hu, Xiang Wang, Zhufa Zhou, Yu Liu

**Affiliations:** 1 College of Chemistry, Chemical Engineering and Materials Science, Soochow University, Suzhou 215023, China; mhu777@stu.suda.edu.cn (M.H.); 20184209228@stu.suda.edu.cn (X.W.); zhouzhufa@suda.edu.cn (Z.Z.); 2 National Engineering Laboratory for Modern Silk, College of Textile and Clothing Engineering, Soochow University, Suzhou 215023, China; liuyu@suda.edu.cn

**Keywords:** europium, mesoporous bioactive glass nanospheres, drug release, osteosarcoma MG 63 cells

## Abstract

Functional ions and drug factors play a vital role in stimulating bone tissue regeneration as we understand it. In this work, europium-containing mesoporous bioactive glass nanospheres (Eu/MBGs), composed of 60% SiO_2_—(36–x)%CaO—x%Eu_2_O_3_—4%P_2_O_5_ (x = 0, 0.5, 1, 2 mol%), were prepared by a facile sol-gel process. The results indicate that Eu ions play an important role to influence the microstructure of MBGs, in which a suitable concentration of Eu (1 mol%) increases their surface area (502 m^2^/g) as well as their pore volume (0.34 cm^3^/g). Proper doping of Eu ions in MBGs can observably induce apatite mineralization and improve the doxorubicin (DOX) release behavior. Furthermore, DOX-loaded Eu/MBGs could maintain a long-term inhibitory effect on the viability of osteosarcoma MG 63 cells. This work has demonstrated that it is possible to develop functional Eu/MBGs by combining excellent apatite-mineralization ability, controllable drug (DOX) release and antitumor functions for the therapy of bone tissue regeneration.

## 1. Introduction

Cancer is one of the major causes of death in China. In 2014, cancer accounted for an estimated 2.3 million deaths, with 3.8 million new cases confirmed, and tumor mortality and incidence rates of 167.89 × 10^5^ and 278.07 × 10^5^, respectively [1]. Some tumors are more frequently associated with bone metastases, such as those related to breast, lung and prostate. Conventional treatments include implant removal followed by radiation and chemotherapeutic drugs that may have limitations and lead to additional toxicity to the patient [2,3]. Therefore, there is urgent need for improving diagnostic means for early detection and for researching more selective drug release systems with few side effects for treatment. Compared with traditional cancer treatment, the development of nano-targeted drug release systems has led to a new strategy because cellular interaction and communication are often at the nano scale [4,5]. The foremost advantages of employing nano drug delivery systems include specific delivery for targeted action, which can overcome the barrier of drug penetration, and enhancement of the bioavailability and therapeutic performance of antitumor drugs.

In recent years, mesoporous bioactive glass nanospheres (MBGs) have demonstrated remarkable properties as carriers for bone tissue regeneration application because of their biodegradability and osteoconductivity [6]. Microstructure properties of biomaterials, such as specific surface area, surface defects and pore texture, play a major role in promoting drug adsorption and release mechanisms. MBGs possess a significantly larger specific surface area and pore volume, therefore, the antitumor drugs can be easily encapsulated, adsorbed, and chemically bound to the glass matrix [7,8]. Furthermore, the physical, chemical and biological properties of MBGs can be made highly tunable with a wide range of biodegradability and biocompatibility features by incorporating different therapeutic ions [9,10,11].

Europium (Eu), located in the middle of the lanthanides in the periodic table, is a biologically active rare earth element with low toxicity, and generally exists in trivalent form with excellent chemical properties [12,13]. In vitro and in vivo toxicity of Eu has been studied, but no systematic research exists into the effect of Eu ions from MBGs on in vitro bioactivity and, in particular, into the in vitro doxorubicin (DOX) release mechanism or the cooperative effect of Eu ions and anti-tumor drug (DOX) from MBGs on the inhibitory effect of osteosarcoma MG 63 cells. The biological properties of Eu are essentially based on their similarity to calcium (Ca) [14,15]. It is well-known that bone is a finely regulated tissue combined with proteins and a mineral phase. The latter is typically recognized as a hydroxyapatite phase, and approximated by the formula Ca_10_(PO_4_)_6_(OH_2_) [16]. Research also indicates that the loaded drug may be easily combined with Ca ions in the MBG materials to tailor the drug’s release mechanism [17]. With a similar ionic radius to Ca, but a higher charge, Eu has a larger ion potential than that of Ca. This makes it more convenient for Eu ions to occupy the Ca sites on biomaterial and thus act as either Ca inhibitors or biochemical probes. Therefore, the aim of this work is to prepare Eu-containing MBG nanospheres (Eu/MBGs) and to investigate the effect of Eu ions from MBGs on their microstructure, bioactivity and DOX release mechanism, as well as the cooperative effect of Eu ions and DOX from MBGs on the inhibitory effect of osteosarcoma MG 63 cells in vitro.

## 2. Material and Methods

### 2.1. Preparation and Characterization of Eu/MBGs

Eu-incorporated MBGs were prepared by incorporating 0, 0.5, 1 and 2 Eu (in mol%) into MBGs following the previously reported method after some modification [18]. A typical synthesis of 1 Eu/MBGs (mole ratio of Si/Ca/Eu/P was 60/35/1/4) was as follows: First, 1.2 g hexadecyltrimethyl ammonium bromide (CTAB) was dissolved in 40 mL ethanol and 60 mL deionized water under stirring at 303 K. Second, 0.8 mL ammonia solution (25 wt%), 3.36 g tetraethyl orthosilicate (TEOS), 2.22 g calcium nitrate tetrahydrate (Ca(NO_3_)_2_·4H_2_O, CN), 0.047 g europium oxide (Eu_2_O_3_), and 0.38 g triethyl phosphate (TEP) were added in turn and the resulting solution was vigorously stirred for another 5.5 h. Then, white precipitate was gathered, washed 3 times with ethanol and deionized water in turn and dried at 333 K for 24 h. Lastly, the gathered white precipitate was calcined at 923 K for 3 h to obtain the 1 Eu/MBGs. Samples of 0 Eu/MBGs, 0.5 Eu/MBGs and 2 Eu/MBGs were prepared using the above method, with differences only in the Eu and Ca content. All the reagents were obtained from Sinopharm Chemical Reagent Co. Ltd (Shanghai, China). 

The surface morphology and structure of the prepared samples were inspected using transmission electron microscopy (TEM, FEI Tecnai G-20, Hillsboro, OR, USA) and scanning electron microscopy (SEM, S-4700, Tokyo, Japan) associated with energy dispersive X-ray spectroscopy (EDS). The local chemical environments of prepared samples were detected by X-ray photoelectron spectroscopy (XPS, ESCALAB 250Xi, Waltham, MA, USA). The specific surface area, pore size and pore volume of prepared samples were determined by N_2_ adsorption-desorption isotherms (BET, ASAP2020 (M+C), Atlanta, GA, USA), and the components of the prepared samples were analyzed by Fourier transform infrared spectroscopy (FTIR, VERTEX 70V, Karlsruhe, Germany), with spectra recorded in the 400–1800 cm^−1^ spectral range with the KBr pellet technique. 

### 2.2. In Vitro Bioactivity Test of Eu/MBGs

Simulated body fluids solution (SBF) was prepared based on the method reported by Kokubo [19]. The order and amounts of reagents for prepared SBF are shown in Table 1. The experimental process was as follows: Eu/MBGs (0.1 g) were incubated in SBF solution (100 mL) at 310 K for 24, 72 and 168 h under shaking at 160 rpm. After each soaking time, Eu/MBGs were removed from SBF, rinsed with deionized water and dried at room temperature. FTIR analyses and SEM micrographs were used to investigate the surface microstructure of the Eu/MBGs.

### 2.3. Drug Release from Eu/MBGs

First, DOX was dissolved in phosphate buffered saline (PBS, pH 7.4, Biosharp, Hefei, China) with concentration of 0.25 mg/mL. Second, Eu/MBGs (50 mg) were soaked into above mixed solution with vibration for 24 h at room temperature. Then, the DOX-loaded Eu/MBGs were separated, washed 3 times and dried at 310 K for 24 h. At the same time, the supernatant was collected. By comparing the difference of concentration before and after DOX loading, the encapsulation efficiency of the DOX-loaded Eu/MBGs could be calculated. Finally, the obtained DOX-loaded Eu/MBGs (Eu/MBGs-DOX) were soaked in 7 mL PBS solution for different periods of time and different pH values (pH 4.3 and pH 7.4) at 310 K for DOX release test. At each time point, 3.5 mL PBS was extracted to test the release of DOX and 3.5 mL of fresh PBS was added back. The extracted medium was analyzed by UV-Vis spectroscopy (UV-Vis, CARY50, Beijing, China) at the characteristic wavelength (λ = 482 nm) [20]. Cumulative release of DOX from Eu/MBGs was counted according to Equation (1) [21]:

(1) $$C_{t,\mathrm{cumu}}=C_{t}+\frac{v}{V}\sum_{0}^{t-1}C_{t}$$

where *C_t,cumu_* is the cumulative release of DOX, *C_t_* is the apparent release at time *t*, *v* is the volume extracted at time *t*, and *V* is the total volume of dissolution medium.

### 2.4. In Vitro Osteosarcoma MG 63 Cells Culture

Human osteosarcoma MG 63 cells were obtained from the Basic Medicine and Life Science Academy of Soochow University in China. The inhibitory effect of Eu/MBGs and Eu/MBGs-DOX on the human osteosarcoma MG 63 cells was evaluated by the CCK-8 method (Sigma, St. Louis, MO, USA) according to the manufacturer’s instructions. MG 63 cells were seeded at a density of 1 × 10^3^ cells/well on the 96-well plates, grown in 0.2 mL of Dulbecco’s modified Eagle’s medium (DMEM, Gibco, Grand Island, USA) containing 10% fetal bovine serum (FBS) and allowed to attach for 12 h in a 5% CO_2_ atmosphere. Then, the graded concentrations of Eu/MBGs and Eu/MBGs-DOX (50, 100 and 200 μg mL^−1^) were added into culture medium. The group without Eu/MBGs or Eu/MBGs-DOX was used as a blank control. After 1, 3 and 7 day incubation respectively, 10 μL of CCK-8 indicator dye (10 mg/mL in PBS, pH 7.4) was added into each well. The absorbance of each well was measured on a microplate reader (Synergy HT, Bio Tek Instruments, Winooski, VT, USA) at 450 nm with a reference wave length of 650 nm. Lastly, the results were obtained in triplicate from 3 separate experiments for each test.

### 2.5. Statistical Analysis

Statistically significant differences between samples were performed using SPSS software. All data are presented as mean ± standard deviation (SD) and carried out using one-way analysis of variance (ANOVA). Difference with *p* < 0.05 (*) or *p* < 0.01 (**) was considered statistically significant.

## 3. Results and Discussion

### 3.1. Morphology and Microstructure of Eu/MBGs

**Figure 1:**
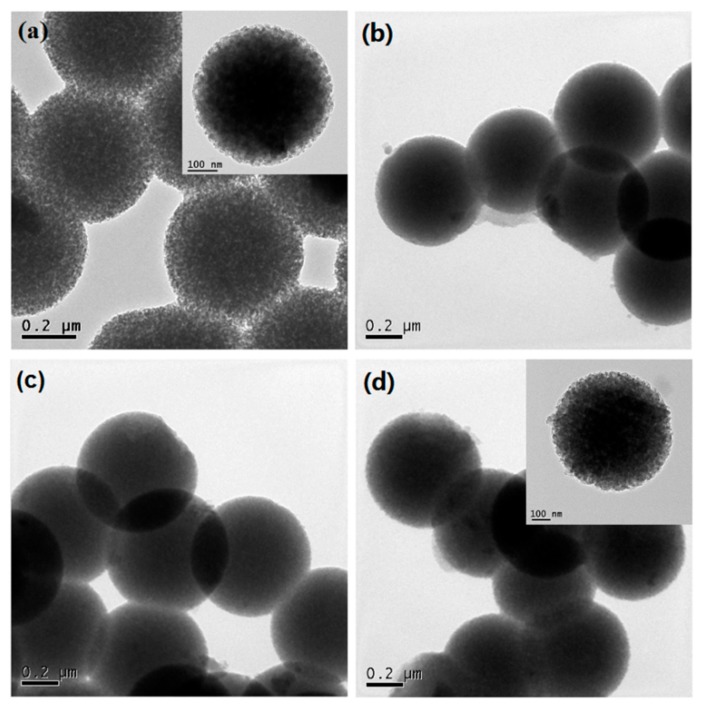
Transmission electron microscopy (TEM) images of 0 europium-containing mesoporous bioactive glass nanospheres (Eu/MBGs) (**a**), 0.5 Eu/MBGs (**b**), 1 Eu/MBGs (**c**) and 2 Eu/MBGs (**d**) (inset HRTEM).

**Figure 2:**
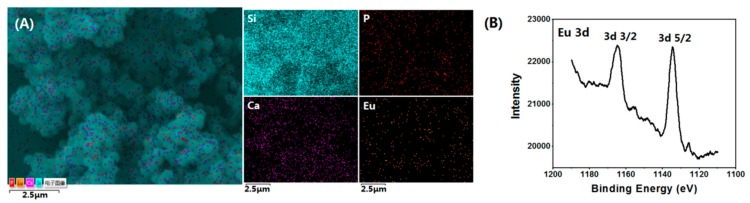
Energy-dispersive X-ray spectroscopy (EDS) mapping for the distribution of Si, P, Ca, and Eu in the 1 Eu/MBGs (**A**), and Eu high resolution X-ray photoelectron spectroscopy (XPS) spectrum of 2 Eu/MBGs (**B**).

**Figure 3:**
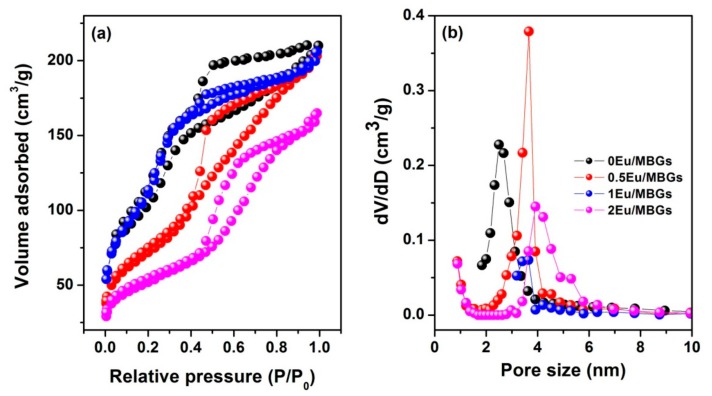
Nitrogen adsorption-desorption isotherm (**a**) and mesopore pore size distribution (**b**) of Eu/MBGs.

Figure 1 shows the surface morphology of Eu/MBGs realizable by a mild sol-gel method using CTAB as a sole template. As shown in Figure 1, all the prepared Eu/MBGs samples took on spherical morphology with uniform particle size. Particle size of the Eu/MBGs was about 500 nm and there were numerous tiny pores inside the nanospheres. Figure 2A displays EDS mapping images of Si, P, Ca, and Eu for 1 Eu/MBGs. The result indicated that all four elements were uniformly distributed throughout the surface of nanospheres, which suggested that the composition of the prepared Eu/MBGs was homogeneous. Figure 2B shows the Eu high-resolution XPS spectrum of 2 Eu/MBGs. The location of peaks for 3d doublets due to spin-orbit coupling, such as d_3/2_ and d_5/2_, are attributed to Eu^3+^ ions around 1164.7 and 1134.5 eV, which proves the existence of Eu [22,23]. Figure 3 indicates that all the BET curves of Eu/MBGs presented a Type IV isotherms pattern with type H1 hysteresis loops, characterizing mesoporous materials [24]. Textural parameters of the Eu/MBGs are listed in Table 2. It can be seen that addition of Eu^3+^ significantly changed BET specific surface area and pore volume of MBGs, with trends in their changes behaving like a parabola. Moreover, 1 Eu/MBGs possesses the highest BET surface area (502 m^2^/g) and pore volume (0.34 cm^3^/g) compared to 0 Eu/MBGs, 0.5 Eu/MBGs and 2 Eu/MBGs.

### 3.2. In Vitro Bioactive Evaluation of Eu/MBGs

**Figure 4:**
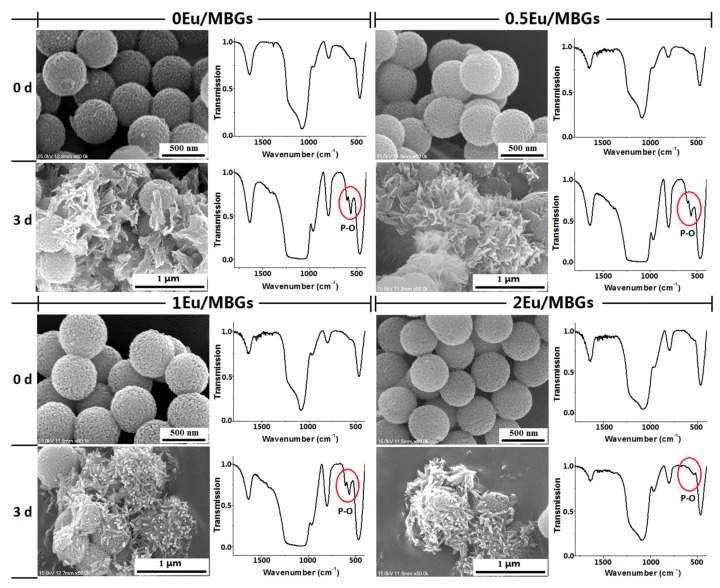
Scanning electron microscopy (SEM) micrographs and Fourier transform infrared (FTIR) spectroscopy analysis of Eu/MBGs after soaking in SBF for 0 and 3 days.

Figure 4 shows FTIR spectra and SEM micrographs of Eu/MBGs after soaking in SBF solution for 0 and 3 days. The scope of the FTIR analysis is on the wave numbers between 400 and 1800 cm^−1^ because the main vibration peaks associated with apatite in the Eu/MBGs samples are in this region. Apatite formation is an important characterization closely related to bioactivity of biomaterials. As exhibited in the FTIR spectra, the 400–1000 cm^−1^ region matches the bands caused by the bending vibrations of Si–O bonds in SiO_4_ tetrahedral, which are centered at ~469 cm^−1^ and ~799 cm^−1^, respectively. After soaking in SBF, the two Si–O bands become sharper. Meanwhile, new peaks assigned to crystalline calcium phosphate appeared at wave numbers of 604 and 563 cm^−1^ in the patterns for 0 Eu/MBGs, 0.5 Eu/MBGs and 1 Eu/MBGs, but not except 2 Eu/MBGs, after soaking in SBF solution for 3 days, indicating the formation of apatite on the surface of Eu/MBGs [25,26]. As shown in the SEM micrographs, coarse spherical surfaces with microscopic pores were observed in all the Eu/MBGs samples before soaking. After soaking for 3 days, some sheet-like apatite is deposited on the surface of Eu/MBGs with low Eu content (0–0.5%). However, with high Eu content (1–2%), some worm-like apatite is found on the surface of Eu/MBGs. It can be observed that the morphology of these mineralized apatite shows noticeable differences among the four Eu/MBGs with different doping Eu content.

Mineralization of apatite depends on the formation of the crystal nucleus and the growth of crystal [27]. When the concentration of phosphorus and calcium in the microenvironment (SBF) reaches the nucleation threshold, the core of the new phase (Ca–P) can be formed on the surface of the Eu/MBGs. Second, by electrostatic attraction or chemical bond, Ca^2+^ and PO_4_^3−^ ions can be adsorbed on the surface of the Eu/MBGs and interact with OH^−^, HPO_4_^2−^ and CO_3_^2−^ ions in SBF, making the apatite crystals grow continuously. Finally, the apatite crystals continue to grow into tiny needle-, sheet-, or rod-shaped crystals and aggregate into globular, petal-shaped clusters or other irregular particles [28,29]. In this study, however, the morphology of apatite changed from sheet to rod with increasing the doping Eu content. The most likely explanation for this is that the large-sized Eu^3+^ (0.115 nm) ion doping results in lattice distortion of Eu/MBGs, which would break the ordered orientation of SiO_4_^4−^. This further hinders the crystallization of apatite and changes its morphology. 

### 3.3. In Vitro Drug Release of Eu/MBGs

**Figure 5:**
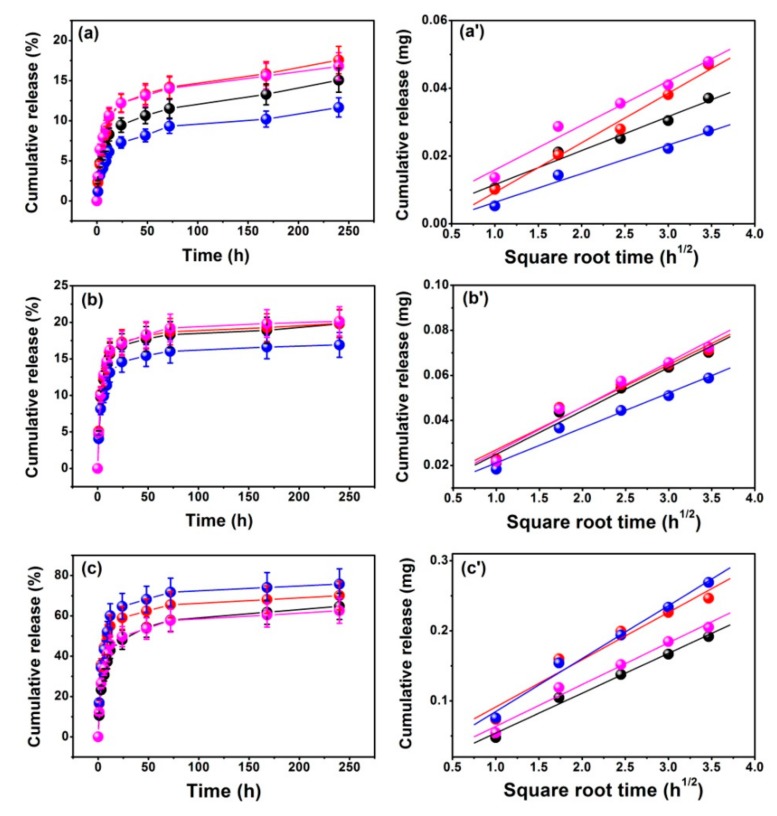
Cumulative release of doxorubicin (DOX) from Eu/MBGs in PBS with different pH values (**a**–**c**) and fitted to the first 12 h of data by the Higuchi model (**a’**–**c’**). (**a**,**a’**) DOX release at pH 8.6, (**b**,**b’**) DOX release at pH 7.4, (**c**,**c’**) DOX release at pH 4.3; black “●” symbols 0 Eu/MBGs; red “●” symbols 0.5 Eu/MBGs; blue “●” symbols 1 Eu/MBGs; magenta “●” symbols 2 Eu/MBGs.

**Figure 6:**
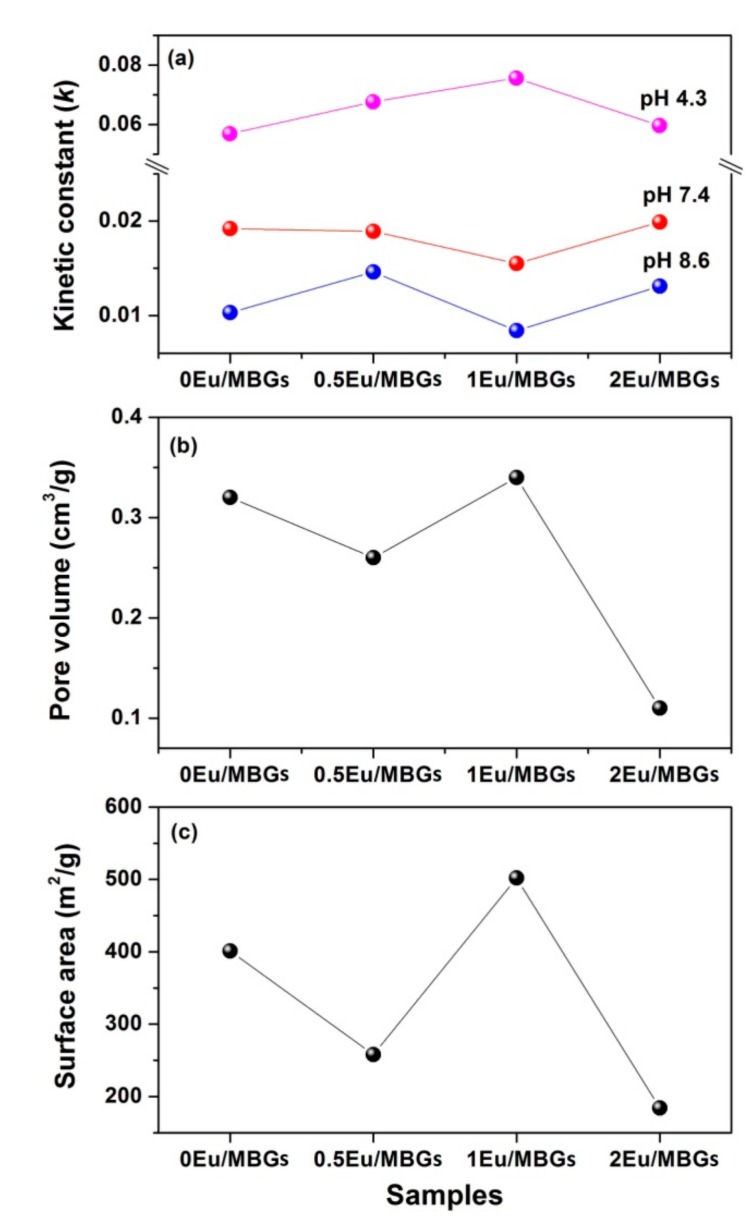
Kinetic constant (**a**) and microstructure parameters (surface area (**b**) and pore volume (**c**)) of Eu/MBGs.

Figure 5a–c presents the DOX release behavior for the Eu/MBGs with different pH values and Eu concentrations. It should be noted that the maximum release value of DOX reached 80% when Eu/MBGs were in PBS at pH 4.3, but only about 20% at pH 7.4 and pH 8.6. Figure 5a’–c’ shows the linear relationship between the amounts of DOX releases and the square root of time in order to evaluate the DOX release mechanism, which illustrated that the DOX release behavior from Eu/MBGs is governed by the Fickian diffusion. These results could be certified by the Higuchi model (Table 3, *Q_H_* = *k_H_* × *t*^1/2^, where *Q_H_* is the drug release amount at time *t* and *k_H_* is the Higuchi constant) [30,31]. The values of kinetic parameters (Table 3) obtained from the Higuchi model indicate that there is a higher DOX release at pH 4.3, and a lower DOX release at pH 7.4 and pH 8.6. The above conclusions can also be drawn as follows: the DOX release behavior follows the sequence of 1 Eu/MBGs > 0.5 Eu/MBGs > 2 Eu/MBGs > 0 Eu/MBGs at pH 4.3; but at pH 7.4, the DOX release behavior follows 2 Eu/MBGs > 0 Eu/MBGs > 0.5 Eu/MBGs > 1 Eu/MBGs; and at pH 8.6, the DOX release rate is 0.5 Eu/MBGs > 2 Eu/MBGs > 0 Eu/MBGs > 1 Eu/MBGs, respectively (Figure 6a). These results show that the suitable Eu content improves the DOX release behavior as well as DOX loading, which is beneficial for therapy of cancer cells that affect the repair of bone defect sites.

As a drug carrier, the characteristics of Eu/MBGs are not only important for bone regeneration and repair, but also play an important part in adsorption and release behavior of the drug. Among these characteristics, specific surface area and pore texture of Eu/MBGs are considered significant features. Logically, high surface area and pore volume are expected to adsorb higher amounts of the drug [32,33]. For example, in this work, the specific surface area of Eu/MBGs from 258 m^2^/g (0.5 Eu/MBGs) to 502 m^2^/g (1 Eu/MBGs), increased their loading capacity of DOX from 1.16 mg to 1.25 mg (Table 2). DOX was chosen as a model drug for its water-soluble characteristics, and the Eu/MBGs had a highly hydrophilic property. Therefore, DOX molecules can easily adsorb on the charged surface and move into the internal pores of prepared Eu/MBGs because of the stronger interaction between nanospheres and DOX. This will facilitate the increase of DOX loading and slow DOX release in PBS (Figure 6a–c). Clearly, the larger specific higher surface areas of Eu/MBGs, the more silanol groups on the surface of 1 Eu/MBGs, allowing more hydroxyl groups (–OH) and amino groups (–NH_2_) to accumulate on the surface of Eu/MBGs by electrostatic interaction [34,35]. Therefore, Eu/MBGs possessed a slower release of DOX than others at higher pH values. With the decrease of the pH value of PBS solution, the surface of 1 Eu/MBGs is positively charged. As a result of the electrostatic repulsion between nanospheres and DOX, the adsorption effect begins to weaken. Hence, 1 Eu/MBGs had a faster release behavior of DOX than others at lower pH values. Based on the above research, it is particularly challenging to fully control all microstructure characteristics of Eu/MBGs for drug release behavior. This conclusion is also applicable to regeneration of such bone substitutes through new bone tissues. 

In conclusion, the overall trend is as follows: the larger the specific surface area and pore volume of the prepared Eu/MBGs, the higher the DOX loading and lower DOX release at higher pH values. In addition, increasing in specific surface area and pore volume of Eu/MBGs is favorable for the accumulation of surface charges, which can influence drug diffusion within the pore texture and thus adjust the adsorption and release behavior of DOX.

### 3.4. In Vitro Osteosarcoma MG 63 Cells Culture Studies

**Figure 7:**
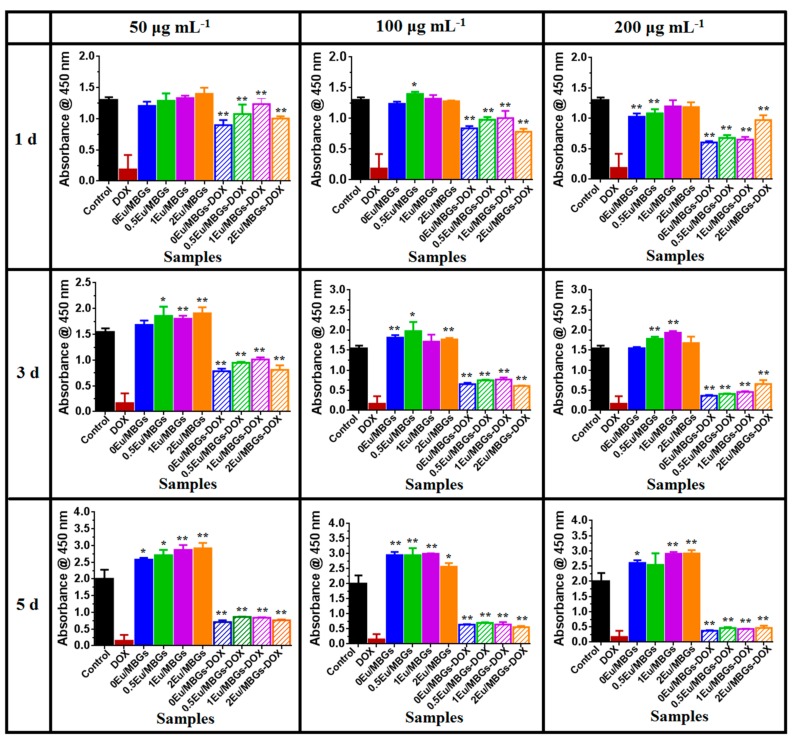
The effects of Eu/MBGs and Eu/MBGs-DOX (50, 100 and 200 μg mL^−1^) on the viability of osteosarcoma MG 63 cells at 1, 3 and 5 days. Data are expressed as the mean ± standard deviation of three independent experiments. *: *p* < 0.05, **: *p* < 0.01.

The viability of osteosarcoma MG 63 cells in the different Eu/MBGs was performed by CCK-8 assay after incubation for 1, 3 and 5 days. There were no significant differences between the cultures among diverse concentrations of Eu/MBGs at 1 day (Figure 7). As shown in Figure 7, with greater culture time, the viability of osteosarcoma MG 63 cells on Eu/MBGs was observably enhanced at 3 and 5 days, especially on the Eu-doped MBGs at concentrations of 50 μg mL^−1^ and 200 μg mL^−1^ (*p* < 0.01). The effect of concentration of Eu doping on MG 63 osteosarcoma cells was not statistically significant. However, the effect of Eu-doped MBGs on cell viability of osteosarcoma MG 63 cells was superior to that of undoped MBGs, regardless of added concentration of nanospheres. A tentative interpretation is that the prepared Eu/MBGs supported the growth of osteosarcoma MG 63 cells.

Meanwhile, the effect of Eu ions and DOX from MBGs on the osteosarcoma MG 63 cell viability was also examined using CCK-8 assay by culturing for 1, 3 and 5 days. According to Figure 7, cell viability of MG 63 decreased gradually with increasing culture time and added concentrations of Eu/MBGs-DOX. Obviously, the Eu/MBGs-DOX possesses a relatively higher cell viability of MG 63 compared with DOX due to the cooperative effect of Eu ions and DOX from MBGs (*p* < 0.01). The results also indicate that the controlled release of DOX delivery from the Eu/MBGs is favorable to maintain a long-term inhibitory effect on the viability of osteosarcoma MG 63 cells.

## 4. Conclusions

In this work, Eu-containing MBGs by a facile sol-gel process were successfully prepared. The microstructure of MBGs was modified with Eu ions, which were selected because of the important part they play in biological functions based on their similarity to calcium. Experimental results showed that the prepared Eu/MBGs have a mesoporous structure, unique apatite-forming ability and controlled drug delivery, which could be efficiently controlled by adjusting the Eu content. Moreover, the Eu/MBGs maintain a long-term inhibitory effect on the viability of osteosarcoma MG 63 cells due to the cooperative effect of Eu ions and DOX. Hence, this kind of bioactive nanosphere with the added values of the dopant is a prospective candidate to use as a nanocarrier in bone tissue engineering.

## Figures and Tables

**Table 1:** Order and amounts of reagents for prepared simulated body fluids solution (SBF).

Order	Reagents	Dose
1	NaCl	8.035 g
2	NaHCO_3_	0.355 g
3	KCl	0.225 g
4	K_2_HPO_4_·3H_2_O	0.231 g
5	MgCl_2_·6H_2_O	0.311 g
6	1.0 M-HCl	39.00 mL
7	CaCl_2_	0.292 g
8	Na_2_SO_4_	0.072 g
9	Tris	6.118 g
10	1.0 M-HCl	0.500 mL

**Table 2:** Textural parameters of the Eu/MBGs.

Samples	Surface Area (m^2^·g^−1^)	Pore Size (nm)	Pore Volume (cm^3^·g^−1^)	Drug Loading (mg)
0 Eu/MBGs	401 ± 0.31	2.49 ± 0.05	0.32 ± 0.01	1.19 ± 0.02
0.5 Eu/MBGs	258 ± 0.50	3.66 ± 0.09	0.26 ± 0.01	1.16 ± 0.03
1 Eu/MBGs	502 ± 1.00	3.50 ± 0.06	0.34 ± 0.01	1.25 ± 0.03
2 Eu/MBGs	184 ± 0.43	3.88 ± 0.08	0.11 ± 0.01	1.12 ± 0.02

**Table 3:** The kinetic constant (*k_H_*), coefficient of determination (*R*^2^) and slope of linear regression values of DOX release from Eu/MBGs with different pH values in vitro.

Samples	0 Eu/MBGs			0.5 Eu/MBGs			1 Eu/MBGs			2 Eu/MBGs		
	pH 4.3	pH 7.4	pH 8.6	pH 4.3	pH 7.4	pH 8.6	pH 4.3	pH 7.4	pH 8.6	pH 4.3	pH 7.4	pH 8.6
*k_H_*	0.0569	0.0192	0.0103	0.0676	0.0189	0.0146	0.0756	0.0155	0.0084	0.0596	0.0199	0.0131
*R* ^2^	0.9949	0.9854	0.9886	0.9751	0.9826	0.9939	0.9936	0.9858	0.9896	0.9910	0.9838	0.9874
*slope*	0.0028	0.0058	0.0015	0.0237	0.0081	0.0053	0.0094	0.0056	0.0021	0.0041	0.0061	0.0028
